# Tongue Necrosis Secondary to Giant Cell Arteritis: A Case Report and Literature Review

**DOI:** 10.1155/2017/6327437

**Published:** 2017-03-23

**Authors:** Rafael Alex Barbosa de Siqueira Sobrinho, Karolina Cayres Alvino de Lima, Helena Carvalho Moura, Mônica Modesto Araújo, Christyanne Maria Rodrigues Barreto de Assis, Pedro Alves da Cruz Gouveia

**Affiliations:** ^1^Internal Medicine Service, Federal University of Pernambuco, Recife, PE, Brazil; ^2^School of Medicine, Federal University of Pernambuco, Recife, PE, Brazil; ^3^Pathology Service, Federal University of Pernambuco, Recife, PE, Brazil

## Abstract

Giant cell arteritis is a form of vasculitis involving the medium- and large-sized arteries that chiefly affects older people. Clinical findings are headache, jaw claudication, fever, pain, and thickening of the temporal artery. The most feared complication is visual loss due to impairment of the ophthalmic artery and posterior ciliary arteries. This a case report of an 85-year-old male presenting with headache and jaw pain, who was admitted with tongue necrosis as an initial manifestation of giant cell arteritis. The necrotic area detached spontaneously after two weeks of therapy with corticosteroids and methotrexate. Reviewing the literature, our patient presented with clinical symptoms consistent with most reports, except for the fact of being male. Although unusual as an initial manifestation, tongue necrosis is an important alert for diagnosing giant cell arteritis. Early diagnosis and treatment of this atypical manifestation may reduce morbidity.

## 1. Introduction

Giant cell arteritis (GCA) is a form of vasculitis involving medium- and large-sized arteries, which mainly affects the extracranial branches of the internal and external carotid arteries, particularly the temporal artery. GCA occurs predominantly in females in a ratio of 1.4 to 3 women for every man and exclusively in patients aged 50 years and over [1, 2]. Common symptoms include headache; visual symptoms such as diplopia, amaurosis fugax, and vision loss; and masticatory muscle changes such as jaw claudication [3, 4].

Early diagnosis is vital in order to avoid complications, such as symptoms of ischemia [5, 6]. In some patients, however, unusual symptoms, such as lingual necrosis may appear as an initial manifestation, thus hindering diagnosis [7–9]. In this report, we present a case of GCA in an elderly male with early tongue necrosis.

## 2. Case Presentation

A male, aged 85 years, arrived in the emergency department having suffered from frontotemporal headache associated with jaw pain over a period of 30 days. He reported that the pain had worsened during the last seven days and that pain had also developed on the floor of the mouth and chin. He also presented with a medical history of hypertension, cataracts, and osteoporosis. On admission, neurological examination and neuroimaging indicated no significant changes.

**Figure 1:**
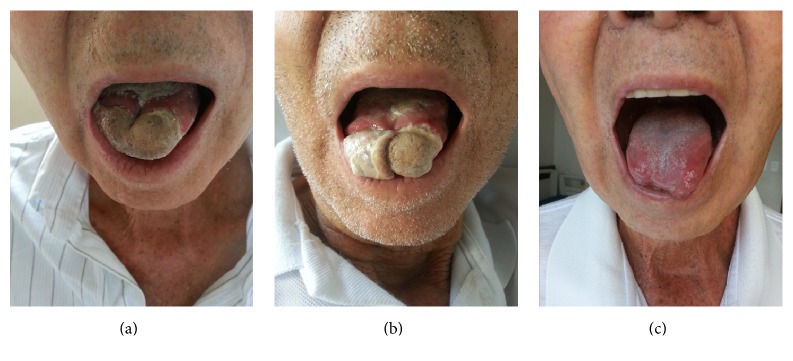
Clinical appearance of the tongue. (a) Tongue infarction at second day. (b) Initial auto-amputation of necrotic tongue at fifth day. (c) Tongue at 20th day presenting full epithelization.

On the second day of hospitalization, the patient presented with increased lingual volume associated with intense pain and difficulty in eating. Inspection of the tongue revealed diffuse edema, whitish plaques, and a small aphthous ulcer. Treatment was initiated for oral candidiasis with fluconazole, and later* Candida dubliniensis* was isolated from a lingual swab. Within 24 hours, the lesion had changed colour, becoming greyish in a bilateral well-defined area, suggesting an ischemic lesion (Figure 1). Temporal pulses were absent and transient bilateral amaurosis appeared, thus confirming the presumptive diagnosis of GCA. Prednisone therapy was initiated with 1 mg/kg/day.

Initial laboratory investigation indicated a complete blood count with mild leukocytosis, neutrophilia, thrombocytosis, and anemia (hemoglobin 11.7 g/dL, hematocrit 36%, 14,290 leukocytes/mm^3^ with 89% neutrophils, and 481,000 platelets/mm^3^). The erythrocyte sedimentation rate (ESR) of 120 mm/h and a C-reactive protein (CRP) of 17.2 mg/dl were consistent with an acute inflammatory process. Other laboratory tests were normal.

**Figure 2:**
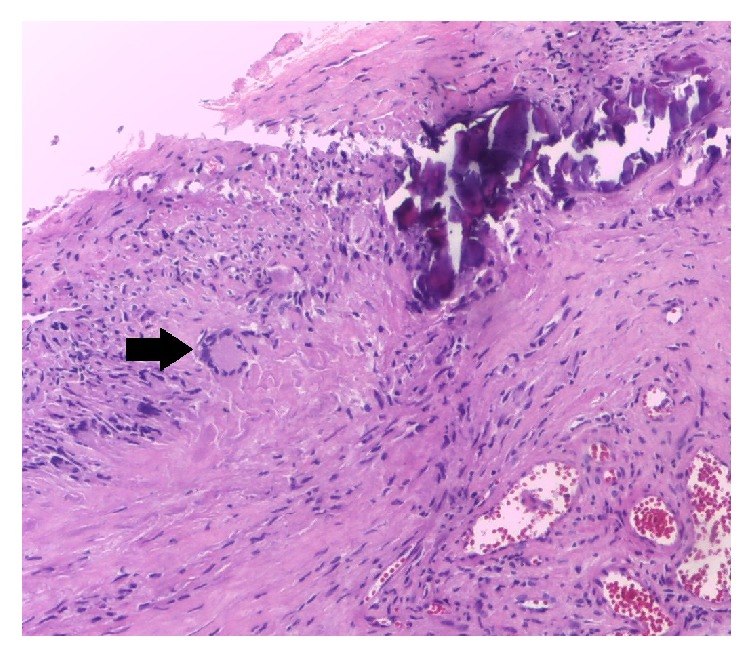
Histological appearance of temporal artery biopsy, showing places of dystrophic calcinosis, extensive transmural infiltrate of lymphocytes, and histiocytes with giant cell (black arrow) placed adjacent to internal elastic lamina (haematoxylin and eosin, original magnification ×100).

Doppler ultrasonography demonstrated that the temporal arteries were tortuous and presented diffuse intimal thickening with edema of the surrounding tissue (halo sign), and stenosis of around 75% of the light from the right temporal artery. A diagnosis of GCA was confirmed with a biopsy from the right temporal artery, which presented partial necrosis of the arterial wall with inflammatory infiltration mainly of mononuclear type (lymphocytes, histiocytes, plasma cells, and multinucleated giant cells) permeating the internal elastic lamina; calcification foci; and total lumen stenosis (Figure 2).

After glucocorticoid therapy was initiated, the patient presented a significant clinical and laboratory response. The area of the tongue that displayed the delimited necrotic lesion detached spontaneously within fourteen days and there was no progression of the ischemic region (Figure 1). After two weeks was initiated methotrexate 10 mg/week, and the patient was discharged clinically stable, and during his outpatient visits no new events or recurrences of GCA were reported. The prednisone dose was initially reduced by 10 mg every month until 20 mg/day and then reduced slowly. After twelve months, with evidence of normal inflammatory activity, the glucocorticoid was completely withdrawn.

## 3. Discussion

The manifestations of GCA include headache in 90% of cases, polymyalgia rheumatica (34%), jaw claudication (50%), amaurosis fugax, and blurred vision (40%). Other findings include fever, increased erythrocyte sedimentation rate, leukocytosis, and abnormalities in the temporal artery [4, 6, 10]. Ocular symptoms should also be highlighted due to the risk of vision loss, for which treatment should be instituted promptly [2].

Lingual manifestations such as edema, pallor, pain, and intermittent claudication occur in up to 25% of cases and can be associated with a greater risk of ischemic complications. However, tongue necrosis is rare, given the rich blood supply to this tissue [1, 11]. GCA with tongue and/or scalp necrosis tends to occur more in older people and develops with more visual symptoms [7]. Tongue necrosis is an unfavorable prognostic sign since it is associated with increased mortality, although not part of the classification criteria [7].

While GCA is the main cause of tongue necrosis, other less common etiologies should be excluded, like carcinoma, embolism, drug use, radiation, syphilis, tuberculosis, chemotherapy, among others [1, 8, 12]. Other causes were not present in this case report; thus treatment was initiated for GCA, since the patient met four of the five criteria cited by the American College of Rheumatology: aged over 50 years, reduced temporal pulse, ESR greater than 50 mm/h, and a compatible biopsy [5, 8, 9].

The use of corticosteroids remains the cornerstone of therapy for GCA [3, 10]. The recommended dose is 1 mg/kg/day of prednisone for four to six weeks and, thereafter, tapering off begins aiming at a dose of 10 mg/day [5]. Recurrences occur in 50% of patients and adverse effects are common [13]. In cases involving visual loss or amaurosis fugax (complicated GCA) intravenous methylprednisolone is indicated for three days before oral glucocorticosteroids. It is possible to try to withdraw corticosteroids after four weeks, paying close attention to clinical symptoms and to ESR and CRP levels [14]. For adjunctive therapy, methotrexate, azathioprine, cyclophosphamide, and anti-TNF-alpha agents may be used [13]. Methotrexate is the first choice as steroid sparing drug because it has proven effective in reducing the cumulative dose of and preventing recurrences [13, 14].

The intensity of the initial inflammatory response of GCA can be determined by five parameters: Platelets > 400,000/mm^3^, temperature > 37.5°C, leukocytes > 11,000/mm^3^, ESR > 100 mm/h, and hemoglobin < 11 g/dl. Patients who meet four or five of these criteria are considered at high risk for recurrence and dependence on corticosteroids [15]. In this case, we chose to initiate methotrexate, given the severity of the recorded criteria (leukocytosis, thrombocytosis, and elevated ESR) associated with tongue necrosis, which in itself presents the worst prognosis. Moreover, the patient presented with comorbidities such as osteoporosis and cataracts, which indicate the need for early use of corticosteroid sparing.

A literature review was conducted using PubMed for case reports either in English or in Spanish of patients presenting with tongue necrosis as a clinical manifestation of GCA. We identified 22 articles published between 2000 and 2015 [1–12, 16–25], which reported 25 cases (Table 1). We found that most patients with GCA-associated lingual necrosis were older females. The ratio was of twelve women for every man, with a mean age of 77 years and an ESR of 79 mm/h. By analyzing the pattern of necrosis, we observed that 80% of patients presented with local pain, 50% with tongue edema, and 28% progressed to ulceration. In relation to associated symptoms, only seven patients (29%) did not present with headache as an initial manifestation. Ocular symptoms, such as blurred vision and sudden visual loss, occurred in 38%. In this review, all patients were treated with high doses of corticosteroids and 28% received pulse therapy with methylprednisolone. The response to corticosteroids was, in the majority (76%) satisfactory, with good healing and disease control. Only one patient progressed to the need for a new course of methylprednisolone [11]. A second drug was used (intravenous cyclophosphamide, methotrexate, and azathioprine) in four cases [6, 11, 12, 25].

For the above-mentioned reasons, our patient presented with clinical findings that were consistent with most reports, except for the fact of being male. Tongue necrosis should serve as a warning for GCA, although it is unusual as an initial manifestation. The physician should be aware of this atypical manifestation, especially since diagnosis and early treatment may change the natural course of the disease, thus reducing morbidity.

## Figures and Tables

**Table 1:** Literature review of giant cell arteritis with tongue necrosis.

Author	Ref.	Age/Sex	ESR	Associated symptoms	Treatment
Mumoli (2015)	[16]	77/F	No	Pain in the scapular cingulum, pain and swelling in both the wrists, transient reduction in visual acuity and headache	Steroids
Zaragoza (2015)	[8]	68/F	55	Moderate headache and swelling of the neck	High dose corticosteroids at 1 mg/kg
Lobato-Berezo (2014)	[17]	74/F	No	Fatigue, anorexia, jaw pain and headache, with ptosis and blurred vision in her left eye	Pulse with methylprednisolone and oral prednisone 1 mg/kg/day
Kumar (2013)	[18]	74/M	132	High-grade fever, bilateral temporoparietal headache, jaw claudication and diminished vision in both eyes	Oral prednisolone 1 mg/kg/day
Grant (2013)	[5]	79/F	68	Sudden complete visual loss in the left eye and generalized ongoing headache	Oral prednisolone 60 mg/day and pulse with 500 mg methylprednisolone for three days
Kumarasinghe (2012)	[19]	74/F	103	Vague history of mild headaches and jaw pain on chewing	Oral prednisolone 40 mg/day
Husein-ElAhmed (2012)	[20]	76/F	87	Generalised weakness	Oral prednisone
Zadik (2011)	[1]	78/F	69	Pain of the right head, neck, face, and shoulder, fatigue, visual blurring and weight loss	Prednisone 60 mg/day
Jennings (2011)	[21]	79/F	75	Fatigue, bilateral occipital neck pain and jaw claudication	Steroid treatment
Olivera (2010)	[4]	74/F	83	Headache, anorexia, weakness and jaw claudication	Oral methylprednisolone 1 mg/kg
Brodmann (2009) Case 1	[7]	81/M	52	Chronic fatigue	Glucocorticoid therapy
Brodmann (2009) Case 2	[7]	79/F	70	Sudden visual loss at the right eye, temporal headache and jaw claudication	High-dose steroid therapy
Zimmermann (2008)	[22]	81/F	69	Bitemporal headache, jaw claudication and mild limb-girdle symptoms	High-dose intravenous hydrocortisone
Schurr (2008)	[10]	66/F	120	Slow speech and a worsening dysphagia	Intravenous prednisone 500 mg for 3 days, then reduced dose of 100 mg
Sainuddin (2008)	[9]	88/F	78	Generalized weakness	Prednisone 40 mg/day
Kusanale (2008)	[23]	86/F	25	No information	Steroids
Ciantar (2008)	[12]	74/F	79	Neck pain	Methylprednisolone 1 g IV for 3 days, prednisolone 60 mg/day and methotrexate 12.5 mg weekly
Goicochea (2007) Case 1	[11]	77/F	40	Asthenia, fever, right temporal headache, and hemifacial pain	Methylprednisolone 1 g IV, cyclophosphamide IV and prednisone 80 mg/day
Goicochea (2007) Case 2	[11]	73/F	42	Frontotemporal headache, arthralgias and lost vision in right eye	Methylprednisolone 1 g IV for 3 days and prednisone 80 mg/day
Goicochea (2007) Case 3	[11]	78/F	125	Headache, left eye visual loss, diplopia, fever and swelling	Methylprednisolone 500 mg IV for 3 days and 60 mg/day orally
Lethert (2007)	[24]	77/F	68	Head, neck and jaw pain, fever, slurred speech and difficulty chewing.	Corticosteroids
Biebl (2004)	[6]	79/F	78	Visual reduction on the left eye, abdominal pain with multiple segmental small bowel necrosis	Prednisolone and azathioprine (100 mg/day each)
García (2003)	[25]	83/F	67	Fever, limb-girdle symptoms, occasional headache	Corticosteroids and methotrexate
Rockey (2002)	[2]	71/F	125	Headache, vasculitic rash in buttocks and ischemia over the distal phalanx of thumb and fifth metatarsals bilaterally	High-dose steroid therapy
Hellmann (2002)	[3]	79/F	115	Fatigue, cough, toothache and visual loss	Methylprednisolone IV in high doses and prednisone 60 mg/day

Ref: reference; ESR: erythrocyte sedimentation rate; M: male; F: female.
